# Red Pitahaya Betacyanins Selectively Upregulate Proinflammatory Cytokines In Vitro During Influenza A Virus Infection

**DOI:** 10.1155/ijm/4807321

**Published:** 2026-10-22

**Authors:** Chie Min Lim, Nurulfiza Mat Isa, Abdul Rahman Omar, Wee Sim Choo

**Affiliations:** ^1^ School of Science Monash University Malaysia Bandar Sunway Selangor Malaysia monash.edu.my; ^2^ Department of Cell and Molecular Biology Faculty of Biotechnology and Biomolecular Sciences Universiti Putra Malaysia Serdang Selangor Malaysia upm.edu.my; ^3^ Laboratory of Vaccines and Biomolecules Institute of Bioscience Universiti Putra Malaysia Serdang Selangor Malaysia upm.edu.my; ^4^ Department of Veterinary Pathology and Microbiology Faculty of Veterinary Medicine Universiti Putra Malaysia Serdang Selangor Malaysia upm.edu.my

**Keywords:** A549 cells, betalain, inflammation, lung epithelial, molecular docking, mRNA, oseltamivir, red dragon fruit

## Abstract

Among the four influenza types, Influenza A virus (IAV) is responsible for annual epidemics and pandemics, causing the most severe illnesses, resulting in patient hospitalizations or death. With IAV threatening the next global influenza pandemic, it is a race against time to search for antiviral drugs. Betacyanins are reddish‐violet, unique water‐soluble nitrogen‐containing pigments that have previously been reported to possess antiviral effects against IAV. The objectives of this study were to study interactions between betacyanins and key inflammatory markers, such as interleukin (IL)‐1*β*, IL‐6, tumor necrosis factor (TNF)‐*α*, and suppressor of cytokine signaling 3 (SOCS3), in IAV‐infected A549 cells. Betacyanins were first extracted from red pitahaya, quantified, and characterized. A549 cells were infected with IAV and then treated with betacyanins. Quantitative PCR was performed to study the change in expression levels of IL‐1*β*, IL‐6, TNF‐*α*, and SOCS3. Molecular docking was performed to study any interactions between betacyanins and IL‐1*β*, IL‐6, TNF‐*α*, SOCS3, and their respective receptors, namely, IL‐1R1, IL‐6R, and TNFR1. Betacyanins modulated IL‐1*β*, IL‐6, and TNF‐*α* uniquely after up to 72 h. Specifically, both IL‐1*β* and IL‐6 were significantly downregulated after 48 and 72 h, and TNF‐*α* was significantly downregulated after 48 h. Additionally, SOCS3, a host factor that IAV hijacks and induces to promote its own survival, was downregulated after 48 and 72 h. In silico analysis showed that betacyanins such as phyllocactin, hylocerenin, betanin, and isobetanin formed binding interactions and hydrogen bonds with IL‐1*β*, IL‐6, TNF‐*α*, SOCS3, IL‐1R1, IL‐6R, and TNFR1. This study suggests that novel interactions between betacyanins and inflammatory cytokines might contribute to the antiviral effects of betacyanins, leading to the survival of the host cell. Future studies into the immunological aspects of the cell and overall cellular health are recommended.

## 1. Introduction

The World Health Organization (WHO) has listed a global influenza pandemic as one of the Top 10 threats to global health in 2019 [1], with the virus causing one billion cases of seasonal influenza annually, including up to 5 million severe cases and up to 650,000 deaths annually [2]. Worryingly, the WHO has warned that a global influenza pandemic is inevitable, with the only uncertainties being its timing and severity [1]. The influenza virus is an enveloped RNA virus that is part of the four classes of the Orthomyxoviridae family, of which currently only IAV and Influenza B virus are clinically relevant to humans. The virus has a high affinity for alveolar epithelial cells in the airway due to the presence of sialic acid residues in the receptors of the epithelial cells [3], leading to significant epithelial damage and leakage of fluids in the airway. The collapse of the airway results in failure of gas exchange, causing hypoxia and respiratory failure. During an IAV infection, one part of the innate immune response that is quickly engaged against the virus to protect the respiratory surface is the inflammatory response, which prevents the replication and spread of the virus [4]. Several proinflammatory cytokines, such as IL‐1*β*, IL‐6, IL‐8, interferon (IFN)‐*α*, IFN‐*β*, and TNF‐*α*, are secreted to promote viral clearance, alongside the recruitment of immune cells, such as neutrophils and macrophages, to the site of infection to prevent the spread of the virus [5].

Despite the importance of these cytokines in mounting a defense against the virus, an overexpression of the cytokines is typically observed in IAV infection, termed cytokine storm, which causes major damage to the lung tissues. Cytokine storm is well documented in clinical settings, is usually associated with the severity of the disease in patients, and is a determinant of patient outcomes [3, 6, 7]. Many in vitro studies have established that infecting human alveolar epithelial cells or macrophages with H1N1, H3N2, or H5N1 viruses leads to a strong induction of inflammatory cytokines such as, but not limited to, TNF‐*α*, IFN‐*α*, IFN‐*β*, IL‐1*β*, IL‐6, and IL‐8 [8]. Additionally, murine models that lacked several inflammatory receptors, such as IL‐1 and TNF receptors, possessed reduced lung inflammation and disease severity after H5N1 infection [9], suggesting the importance of controlled cytokine release.

Betacyanins are reddish‐violet, water‐soluble nitrogen‐containing pigments belonging to the class of pigments called betalains. Relatively speaking, betacyanins are less commonly found in nature compared with other plant pigments such as chlorophylls, anthocyanins, and carotenoids [10]. The Amaranthaceae (namely, *Beta vulgaris* L. and *Amaranthus* sp.) and Cactaceae (namely, *Opuntia* sp. and *Hylocereus* sp.) families are two of the most common and well‐known sources of betalains [10]. Betacyanins have been shown to exhibit antimicrobial activity against a broad range of microbes, most notably against multidrug‐resistant bacteria and biofilm‐producing bacteria [11]. A previous study has reported antiviral properties of betacyanins from red pitahaya (*Hylocereus polyrhizus*) against IAV [12].

Betacyanins can downregulate the expression levels of many proinflammatory cytokines in numerous in vitro and in vivo models, clinical settings, and cancer models, potentially leading to a more positive therapeutic outcome. On the in vitro scale, bone marrow–derived macrophages from mice and primary microglial cells treated with lipopolysaccharide (LPS) have an upregulation of TNF‐*α*, IL‐1*β*, and IL‐6 at the protein level and TNF‐*α*, nitric oxide Synthase 2 (NOS2), interferon regulatory Factor 7 (IRF7), and nuclear factor‐kappa B (NF‐*κ*B) at the mRNA level. Pretreatment and cotreatment with betanin purified from red beetroot before LPS treatment reversed all the abovementioned cytokines after at least 24 h [13–15]. Next, on the in vivo scale, rats with paraquat‐induced acute lung injury presented upregulation of IL‐1*β* and TNF‐*α*, with downregulation of IL‐10, leading to swollen and congested lungs. Rats that received 25 or 100 mg/kg of betanin from red beetroot reversed the up‐ or downregulation of the abovementioned cytokines, with a more positive treatment outcome [16]. Mice with rotenone‐induced Parkinson′s disease unsurprisingly exhibited worse locomotor functions than the control group, as well as increased levels of TNF‐*α*, IL‐1*β*, and IL‐6. The treatment group that received 50 and 100 mg/kg of betanin daily for 18 days restored motor functions similar to the control group and suppressed the levels of TNF‐*α*, IL‐1*β*, and IL‐6 [17]. A clinical trial involving Type 2 diabetic patients gave 12 mL of concentrated beetroot juice twice daily for 12 weeks. At the end of the trial, the levels of IL‐6, TNF‐*α*, and NF‐*κ*B in the patients′ serum were significantly reduced, suggesting a positive therapeutic outcome [18]. Lastly, betanin was also able to significantly downregulate the expression levels of IL‐1*β*, IL‐6, TNF‐*α*, NF‐*κ*B, and Cyclooxygenase 2 (COX2) in cancer models such as human oral squamous cancer cells SCC131 and SCC4 [19] and mice with colon cancer [20] without additional induction from compounds such as LPS or paraquat, as seen from the in vitro and in vivo examples.

As mentioned, IAV infection is usually associated with an overexpression of proinflammatory cytokines, leading to damage to the host cells and tissues. Many studies have suggested that betacyanins can reduce the expression levels of proinflammatory cytokines in nonviral models. However, no such studies have been performed on viral models. Therefore, the present study is aimed at investigating the ability of betacyanins to modulate the expression levels of several key cytokines—IL‐1*β*, IL‐6, and TNF‐*α*, as well as a key cytokine regulator that IAV induces for its own viral replication, SOCS3. It is hypothesized that betacyanins can modulate the expression levels of these cytokines.

## 2. Methods

### 2.1. Betacyanin Extraction, Semipurification, Quantification, and Characterization

Red pitahaya (*H. polyrhizus*) fruits were purchased from a local supermarket (Aeon, Malaysia). A voucher specimen (Specimen Number KLU50213) of the fruits was deposited at Rimba Ilmu Botanic Garden, Universiti Malaya, Selangor, Malaysia. A refractometer (Atago, Japan) was used to determine the total soluble solids (°Brix) of the fruits. Fruit flesh between 10°Bx and 14°Bx was chosen for downstream extraction, semipurification, quantification, and characterization. The extraction process was performed according to the method of Lim et al. [12]. Semipurification of the extracted crude betacyanins was performed as described by Chang et al. [21] and the product is termed the betacyanin fraction. Betacyanin content was quantified and characterized via high‐performance liquid chromatography (HPLC) and liquid chromatography–tandem mass spectrometry (LC‐MS/MS) according to the method of Lim et al [12].

### 2.2. Cell Culture

The A549 human lung adherent epithelial cell line (CCL‐185, ATCC, United States; RRID: CVCL_0023) was routinely maintained in high‐glucose Dulbecco′s Modified Eagle Medium (DMEM; Gibco, United States) supplemented with 10% fetal bovine serum (Tico Europe, Netherlands), without the use of antibiotics. Cells were cultured in a 37°C humidified incubator supplied with 5% CO_2_ and passaged as required. Routine screening for *Mycoplasma* contamination was performed using a polymerase chain reaction (PCR)–based method with primer pairs as described by another study [22].

### 2.3. Virus Infection

A549 cells were infected with Influenza A virus (H1N1) strain A/PR/8/34 (ATCC, United States) according to the methods of Lim et al. with slight modifications. One modification was to provide the betacyanin‐treatment group with only 50.0 *μ*g/mL of betacyanin fraction, as previous data indicated this concentration possessed the highest level of antiviral activity while not exhibiting significant cytotoxicity to A549 cells [12]. Additionally, a positive control group was established by supplementing the virus growth media with an additional 100 *μ*M of oseltamivir phosphate (FluHalt, India) [9]. Moreover, all treatment groups were incubated for 24, 48, or 72 h at 37°C. After incubation, TRIzol reagent (Invitrogen, United States) was added to the cells. The A/PR/8/34 strain was used because it infects the A549 cell line well at a multiplicity of infection of 1 [23, 24].

### 2.4. Human Inflammatory Marker mRNA Expression Analysis

Total RNA was extracted using the TRIzol reagent following the manufacturer′s protocol with slight modifications. The only modification made was replacing the water used to solubilize the RNA pellet with a buffer containing 20 mM dithiothreitol (Vivantis, Malaysia) and 1 mM sodium citrate (Merck, Germany). RNA integrity was determined by agarose gel electrophoresis, and RNA concentration and purity were assessed using a BioDrop Duo spectrophotometer (Biochrom, United Kingdom). All collected RNAs were stored at −80°C until further use.

Complementary DNA (cDNA) synthesis was performed using the ReverTra Ace qPCR RT Master Mix with gDNA Remover kit (Toyobo, Japan), following the manufacturer′s protocol. The resulting cDNA was stored at −20°C until use.

Quantitative PCR (qPCR) was performed using the PrecisionPLUS qPCR Master Mix (Primerdesign, United Kingdom), following the manufacturer′s instructions. Amplification was performed on the CFX96 Touch Real‐Time PCR Detection System (Bio‐Rad, United States; RRID: SCR_018064) under the following cycling conditions: 95°C for 2 min, followed by 40 cycles of 95°C for 10 s and 60°C for 60 s. Data were collected at the end of each cycle, and a melt curve analysis was performed postamplification to confirm the specificity of the PCR products. A nontemplate control (NTC) was included to verify the absence of contamination. Data analysis was performed using the CFX Maestro Software (Bio‐Rad, United States). The primer sequences used in this assay are found in Table S1.

### 2.5. In Silico Analysis

In silico analysis was performed via molecular docking between betacyanins and selected host factors to investigate their interactions. Existing protein models were screened via SWISS‐MODEL (RRID: SCR_018123) [25]. The model quality was evaluated via the Ramachandran plot through PDBsum (RRID: SCR_006511) [26]. The 3D structures of several betacyanins were acquired from PubChem. A blind‐docking approach between the betacyanins and IL‐1*β*, IL‐6, TNF‐*α*, SOCS3, IL‐1R1, IL‐6R, and TNFR1 was carried out using AutoDock (Version 4.2.6, the Scripps Research Institute, United States; RRID: SCR_012746). The protein–ligand interactions were visualized and examined using BIOVIA Discovery Studio Visualizer (Version 21.1.0.20298, Dassault Systèmes, France; RRID: SCR_015651). The Simple Modular Architecture Research Tool (SMART) was used to identify key domains in the studied host factors and the respective receptors [27]. The lowest binding energy levels between the protein and ligand, and any hydrogen bond formations, were determined.

### 2.6. Data Analysis

All experiments were performed in biological triplicate. The fold change in human inflammatory marker mRNA expression between groups was analyzed using the delta‐delta Ct method [28]. All data were expressed as mean value ± standard deviation. Data collected from qPCR were analyzed using two‐way ANOVA and Tukey post hoc test using GraphPad Prism (Version 9, GraphPad, United States; RRID: SCR_002798) to determine statistical significance between groups. A *p* value less than 0.05 was considered significant.

## 3. Results and Discussion

### 3.1. Betacyanin Modulated mRNA Expression Levels of IL‐1*β*, IL‐6, and TNF‐*α* After 48 and 72 h

Figure 1 shows the mRNA expression levels of three inflammatory cytokines, IL‐1*β*, IL‐6, and TNF‐*α*, in IAV‐infected A549 cells after treatment with 50 *μ*g/mL of betacyanin fraction for 24, 48, and 72 h. Firstly, no significant differences were observed between any treatment groups for all three cytokines after 24 h. At the 48‐ and 72‐h mark, both IL‐1*β* and IL‐6 exhibited similar patterns, whereby betacyanin treatment caused a significant upregulation of both cytokines compared with the virus‐only control group. For the case of IL‐1*β*, compared with the virus‐only group, there was a significant upregulation of 81.9‐fold (*p* value: 0.0029) and 69.1‐fold (*p* value: 0.0128) after 48 and 72 h, respectively. Whereas on the side of IL‐6, compared with the virus‐only group, there was a significant upregulation of 1256‐fold (*p* value: < 0.0001) and 911‐fold (*p* value: < 0.0001) after 48 and 72 h, respectively. With regard to TNF‐*α*, betacyanins significantly upregulated the expression of TNF‐*α* by 322.8‐fold (*p* value: 0.0003) after 48 h and then significantly downregulated its expression by 195.8‐fold (*p* value: 0.0291) after 72 h. Treatment with 100 *μ*M of oseltamivir in infected cells exhibited no significant changes in expression levels of IL‐1*β* and IL‐6 when compared with the virus‐only group. Still, it exhibited a downregulation of TNF‐*α* by 278.4‐fold (*p* value: 0.0014) after 72 h.

**Figure 1:**
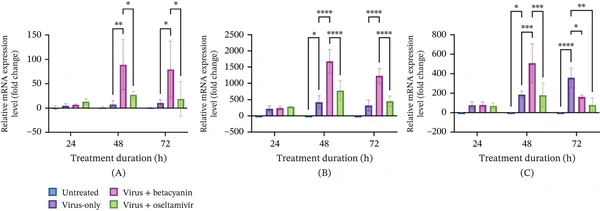
Relative mRNA expression levels (fold change) of (A) IL‐1*β*, (B) IL‐6, and (C) TNF‐*α* in IAV‐infected A549 cells following treatment with 50 *μ*g/mL of betacyanin fraction at 24, 48, and 72 h postinfection. Results are presented as mean ± standard deviation (*n* = 3). Significant differences between groups are indicated as follows:  ^∗^ *p* < 0.05,  ^∗∗^ *p* < 0.01,  ^∗∗∗^ *p* < 0.001, and  ^∗∗∗∗^ *p* < 0.0001.

Intriguingly, the upregulation of both IL‐1*β* and IL‐6 by betacyanin was not reported in any literature, as the current literature only suggested the downregulation of both IL‐1*β* and IL‐6 in numerous in vitro and in vivo nonviral models, as well as clinical trials [13–20]. Similarly, the upregulation of TNF‐*α* after 48 h was also not reported in any betacyanin literature. The present data seemingly contradict the current literature, where betacyanin treatment of infected cells led to an upregulation of proinflammatory cytokines. As mentioned, it is well‐established that during an IAV infection, numerous inflammatory cytokines are produced, and as such, much literature has suggested the importance of targeting the expression of such cytokines and achieving a potential therapeutic effect [29–31]. However, the role of inflammatory cytokines has presented itself as a double‐edged sword—on the one hand, an increased level can cause excessive inflammation, contributing to increased lung injury due to disruptions of the alveolar surface tension, resulting in the manifestation of clinical symptoms. But at the same time, they contribute to immunity against the virus and viral clearance [32]. Data have shown that IL‐1*β*, IL‐6, and TNF‐*α* have been reported to possess strong antiviral capabilities and are crucial in contributing to viral clearance and potentially decreasing mortality [33–37]. Additionally, previous data have demonstrated that betacyanins possess antiviral effects against IAV by significantly reducing virus titer up to 72 h and reducing cytopathic effect (CPE) formation up to 48 h upon 50 *μ*g/mL betacyanin fraction treatment [12]. For instance, 12.5, 25.0, and 50.0 *μ*g/mL of betacyanin fraction significantly reduced the virus titers of IAV‐infected A549 cells by 23.6%, 22.6%, and 34%, respectively, after 24 h of betacyanin treatment. After 48 and 72 h, 50 *μ*g/mL of betacyanin fraction further reduced virus titers by 24% and 43%, respectively [12]. The results from this study, combined with previous data, further suggest that the upregulation of IL‐1*β*, IL‐6, and TNF‐*α* contributed to the survival of the infected cell in the form of antiviral effects rather than to the detriment of the cell. Betacyanin fractions at 50 *μ*g/mL or less have also been shown not to exhibit significant cytotoxicity to A549 cells, as shown by previous data, which demonstrates its safety profile for use on A549 cells at betacyanin fractions of 50 *μ*g/mL or less [12]. In nonviral models, other phytochemicals like polyporus polysaccharide were found to possess anticancer properties against bladder cancer by upregulating and increasing the secretion of proinflammatory cytokines like IL‐1*β*, IL‐6, and TNF‐*α* in macrophages and polarizing the macrophages to the M1 form, achieving anticancer effects [38, 39]. Whereas these studies were performed on nonviral models, macrophages are essential for protection against IAV infection [40]. Regardless, future studies, such as investigations of the host cell′s cellular health, IFN‐*α* and IFN‐*β* secretion, and cytokine protein expression, could help complement the observed changes in the expression levels of IL‐1*β*, IL‐6, and TNF‐*α*.

### 3.2. Betacyanin Downregulated mRNA Expression Levels of SOCS3 After 48 and 72 h

Figure 2 shows the mRNA expression level of SOCS3 in IAV‐infected A549 cells after treatment with 50 *μ*g/mL of betacyanin fraction for 24, 48, and 72 h. Similar to IL‐1*β*, IL‐6, and TNF‐*α*, there were no observable differences after 24 h. After 48 and 72 h, IAV infection led to a significant upregulation of SOCS3 by 14.3‐fold (*p* value: < 0.0001) and −10.7‐fold (*p* value: < 0.0001), respectively, when compared with the untreated control. Treatment of infected cells with 50 *μ*g/mL of betacyanin fraction led to a significant downregulation of SOCS3 by 5.6‐fold (*p* value: 0.0261) and 6.7‐fold (*p* value: 0.0071) after 48 and 72 h, respectively, when compared with the infected‐only control. Similarly, 100 *μ*M of oseltamivir also led to a significant downregulation of SOCS3 by 10.7‐fold (*p* value: < 0.0001) and 9.2‐fold (*p* value: 0.0002) after 48 and 72 h, respectively, when compared with the virus‐only control.

**Figure 2:**
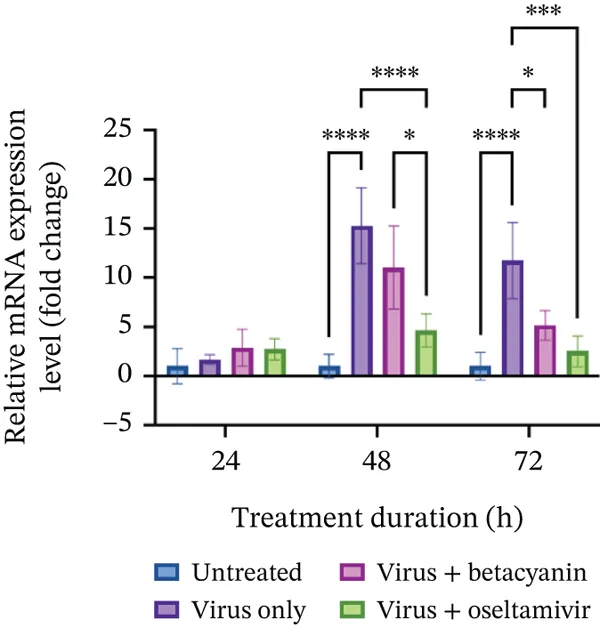
Relative mRNA expression levels (fold change) of SOCS3 in IAV‐infected A549 cells following treatment with 50 *μ*g/mL of betacyanin fraction at 24, 48, and 72 h postinfection. Results are presented as mean ± standard deviation (*n* = 3). Significant differences between groups are indicated as follows:  ^∗^ *p* < 0.05,  ^∗∗∗^ *p* < 0.001, and  ^∗∗∗∗^ *p* < 0.0001.

SOCS3 is a well‐known host factor that the virus hijacks and upregulates for its benefit by suppressing antiviral cytokines in the form of an IL‐6 surge and inhibition of Type I IFN signaling [41–43]. The literature has reported that SOCS3‐deficient mice and SOCS3 antagonists possessed increased protection against damage done by IAV [41, 44]. The present data are in accordance with current literature, where IAV infection led to an upregulation of SOCS3 and IL‐6. Interestingly, whereas betacyanin treatment led to a downregulation of SOCS3 after 48 and 72 h, levels of IL‐6 remained higher than the virus‐only control (Figure 1). To the authors′ best knowledge, this is the first time that betacyanin showed interactions with SOCS3. Thus, it is postulated that there are unique and novel interactions between betacyanin and the host factors in IAV‐infected cells, where the induction of IL‐6 despite the reduction in SOCS3 is beneficial to the cell′s survival in the form of an antiviral defense mechanism against IAV. As mentioned, previous data have shown that 50 *μ*g/mL of betacyanin fraction exhibited significant antiviral effects against IAV by reducing viral titers and CPE formation after up to 72 h [12]. Outside of viral models, several natural compounds such as flavonoid‐rich extract from *Gongronema latifolium*, anthocyanin from *Aronia melanocarpa*, and quercetin were able to ameliorate Type 2 diabetes in both in vitro and in vivo models by targeting SOCS3, resulting in positive therapeutic outcomes [45–47]. Additionally, to the authors′ best knowledge, this is the first time a study was performed, albeit not intentionally, that showed the effects of oseltamivir phosphate on IAV‐infected A549 cells on various inflammatory markers.

### 3.3. Binding Energy Profiles of Betacyanins Against IL‐1*β*, IL‐6, TNF‐*α*, SOCS3, and Their Respective Receptors

Molecular docking is an in silico, structure‐based approach widely used in drug discovery to predict interactions between ligands and targets at the molecular level [48]. The present study examined the lowest binding energy profile between betacyanins and IL‐1*β*, IL‐6, TNF‐*α*, and SOCS3, as well as their respective receptors, namely, IL‐1R1, IL‐6, and TNFR1. Tables 1 and 2 show the lowest binding energy values of selected betacyanins (phyllocactin, hylocerenin, betanin, and isobetanin) against these four host factors and their receptors, respectively, and Tables 3 and 4 show the hydrogen bond formations between the selected betacyanins and the amino acids in the host factors and their receptors, respectively. Figures 3, 4, 5, and 6 represent the two‐dimensional poses of betacyanins docked against the host factors and their receptors. Phyllocactin (57.1%), hylocerenin (12.2%), betanin (22.2%), and isobetanin (4.7%) were identified in the red pitahaya [9] used in this study. Therefore, these betacyanins were selected for molecular docking. Other betacyanin in silico studies have indicated that studying binding interactions between betanin and other host factors, such as Sirtuin 1 [49], NF‐*κ*B [17], and acetylcholinesterase [50], is helpful and useful in drug discovery by observing binding affinities between ligands and proteins, as the binding affinities between betanin and the abovementioned proteins might explain the observed experimental changes in expression levels after betanin treatment.

**Table 1:** The lowest binding energy between various betacyanin types and IL‐1*β*, IL‐6, TNF‐*α*, and SOCS3.

Target	Lowest binding energy (kcal/mol)			
	Phyllocactin	Hylocerenin	Betanin	Isobetanin
IL‐1*β*	−10.49	−10.38	−13.64	−11.23
IL‐6	−11.13	−12.35	−11.97	−10.12
SOCS3	−14.89	−12.86	−13.61	−12.21
TNF‐*α*	−9.49	−7.19	−11.32	−10.21

**Table 2:** The lowest binding energy between various betacyanin types and IL‐1R1, IL‐6R, and TNFR1.

Target	Lowest binding energy (kcal/mol)			
	Phyllocactin	Hylocerenin	Betanin	Isobetanin
IL‐1R1	−7.01	−8.34	−8.93	−8.22
IL‐6R	−6.80	−7.38	−10.84	−9.83
TNFR1	−8.64	−6.65	−10.17	−10.20

**Table 3:** Hydrogen bond formations between various betacyanin types and amino acids in IL‐1*β*, IL‐6, TNF‐*α*, and SOCS3. Brackets indicate how many hydrogen bonds were formed. Bolded amino acids represent crucial residues responsible for protein activity.

Target	H‐bond interaction			
	Phyllocactin	Hylocerenin	Betanin	Isobetanin
IL‐1*β*	**THR195** (2), **VAL248** (2)	**GLU141**, **LYS190**, **THR195**, **LEU196**, **PRO247**, **ASP258**	**GLU141**, **ASP191**, **LEU196**, **VAL248** (2)	**GLU153** (2), **GLN154**, **GLN155**, **VAL157**, **PHE266** (2)
IL‐6	LEU92 (2), GLU121, TYR125, **ASN172** (2)	GLU87, LEU92 (2), LYS94, GLU121 (2), **LEU175**, **LYS178**	GLU121 (2), SER197, GLU200	LEU92, LYS94, LYS114, **THR171**
SOCS3	**SER106**, **SER110**, **THR111**, TYR168 (2)	TYR166, TYR168 (2), SER169, GLY171	**ARG94**, **GLN105**, **SER110**	**THR111**, ARG162 (4), TYR166
TNF‐*α*	**GLU99**, **GLY100**, **LYS141**, **ARG214**, **PRO215**, **ASP219**	**PRO176** (2), **GLU186**, **PRO189**, **TYR191**	**GLN143** (2), **GLY144**, **PRO215**, **TYR217** (2), **ASP219**	**PRO96**, **GLN143**, **LEU218**

**Table 4:** Hydrogen bond formations between various betacyanin types and amino acids in IL‐1R1, IL‐6R, and TNFR1. Brackets indicate how many hydrogen bonds were formed. Bolded amino acids represent crucial residues responsible for protein activity.

Target	H‐bond interaction			
	Phyllocactin	Hylocerenin	Betanin	Isobetanin
IL‐1R1	**LYS157** (3), **TRP160**, **LEU167**, **LEU169**, **SER199**	**ASP256** (2), **ILE267**, **GLU276** (2)	**VAL33**, GLN125 (3)	GLN125 (2), PHE128 (1), **TYR144**, **GLU146** (2)
IL‐6R	**PRO218**, **PRO219**, **ILE222** (2), ALA310	**ASN221**, **ILE222**, **ARG287**, GLU309, ALA310, MET311 (2)	**PRO218** (2), GLU309, MET311	**LYS271**, **GLN274**, **HIS276**, **HIS280**, **ASP281**
TNFR1	**LYS121**, **ASN123**, **GLU136** (2)	**SER61** (2), **ARG66** (2), ASP82, **CYS85**	**CYS87** (2), **GLN91**, **SER107**, **CYS118** (2)	**TYR92**, **ASN105**, **LEU108**, **LEU110**, **ASN123**, **ASN137**, **CYS139** (2)

**Figure 3:**
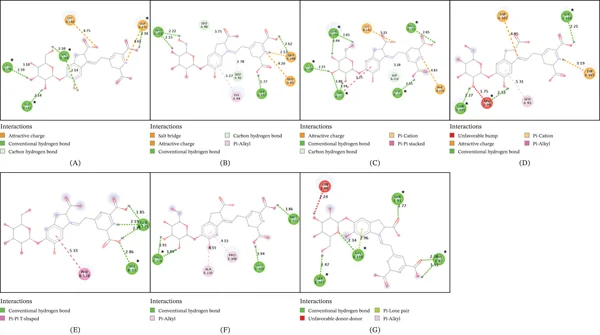
Two‐dimensional molecular docking interaction poses of betanin docked against (A–D) host factors and (E–G) their respective receptors: (A) IL‐1*β*, (B) IL‐6, (C) TNF‐*α*, (D) SOCS3, (E) IL‐1R1, (F) IL‐6R, and (G) TNFR1. Values seen in each pose represent the bond length between the interacting amino acid residues and betanin. The interaction types depicted in each pose are described in the legend below the respective panel. Notably, the green dotted lines represent hydrogen bonds. Crucial amino acid residues were labeled with an asterisk ( ^∗^).

**Figure 4:**
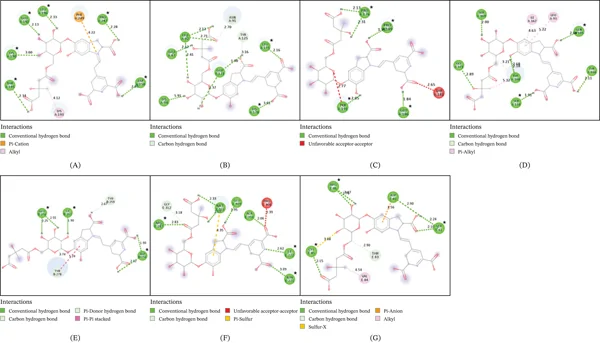
Two‐dimensional molecular docking interaction poses of hylocerenin docked against (A–D) host factors and (E–G) their respective receptors: (A) IL‐1*β*, (B) IL‐6, (C) TNF‐*α*, (D) SOCS3, (E) IL‐1R1, (F) IL‐6R, and (G) TNFR1. Values seen in each pose represent the bond length between the interacting amino acid residues and hylocerenin. The interaction types depicted in each pose are described in the legend below the respective panel. Notably, the green dotted lines represent hydrogen bonds. Crucial amino acid residues were labeled with an asterisk ( ^∗^).

**Figure 5:**
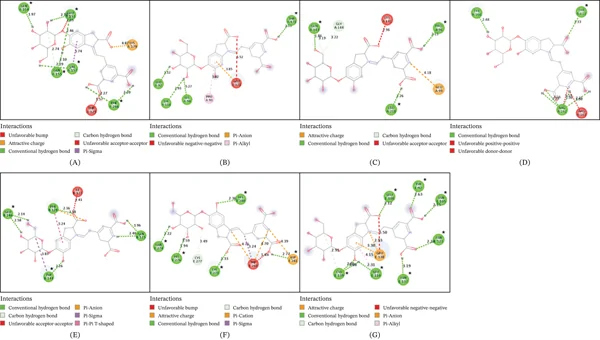
Two‐dimensional molecular docking interaction poses of isobetanin docked against (A–D) host factors and (E–G) their respective receptors: (A) IL‐1*β*, (B) IL‐6, (C) TNF‐*α*, (D) SOCS3, (E) IL‐1R1, (F) IL‐6R, and (G) TNFR1. Values seen in each pose represent the bond length between the interacting amino acid residues and isobetanin. The interaction types depicted in each pose are described in the legend below the respective panel. Notably, the green dotted lines represent hydrogen bonds. Crucial amino acid residues were labeled with an asterisk ( ^∗^).

**Figure 6:**
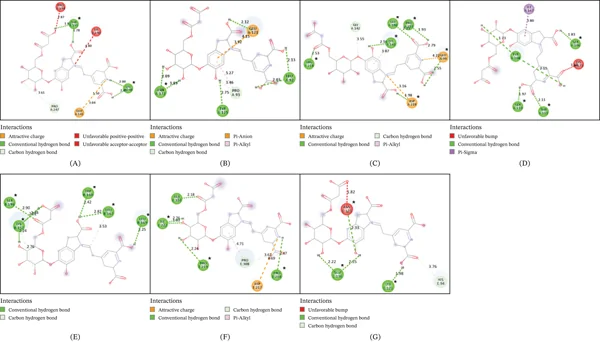
Two‐dimensional molecular docking interaction poses of phyllocactin docked against (A–D) host factors and (E–G) their respective receptors: (A) IL‐1*β*, (B) IL‐6, (C) TNF‐*α*, (D) SOCS3, (E) IL‐1R1, (F) IL‐6R, and (G) TNFR1. Values seen in each pose represent the bond length between the interacting amino acid residues and phyllocactin. The interaction types depicted in each pose are described in the legend below the respective panel. Notably, the green dotted lines represent hydrogen bonds. Crucial amino acid residues were labeled with an asterisk ( ^∗^).

As observed in Figures 3, 4, 5, and 6, the strength of the hydrogen bonds formed between the four betacyanins and the host factors or the receptors generally falls in the range of moderate to strong based on the bond lengths, which contributed to the binding energy levels observed (Tables 1 and 2) and the stability of the protein–ligand complex formed. In general, hydrogen bond lengths between 2.2 and 2.5 Å are considered strong, and 2.5 and 3.2 Å are considered moderate [51]. Their differences in chemical structure can explain the difference in binding energy between betacyanins. In the basic betacyanin structure, different R groups on the *cyclo*‐DOPA moiety give rise to different betacyanins (Figure 7). Betanin, hylocerenin, and phyllocactin have a *β*‐glucose, 3‐hydroxy‐3‐methylglutaryl, and 6 ^′^‐O‐(malonyl)‐*β*‐glucose as one of their R groups, respectively (Figure 8). Isobetanin, on the other hand, is an isomer of betanin. Additionally, whereas hydrogen bonds generally play the biggest role in determining binding energy due to their ability to stabilize a ligand at a targeted protein structure [52], other interactions, such as salt bridges, pi–pi interactions, and sulfur bonds, can contribute to the overall binding energy as well (Figures 3, 4, 5, and 6). For example, the binding energy difference for IL‐6 between hylocerenin and betanin was only 0.38 kcal/mol (Table 1), despite the number of hydrogen bonds formed being eight and four, respectively (Table 3).

**Figure 7:**
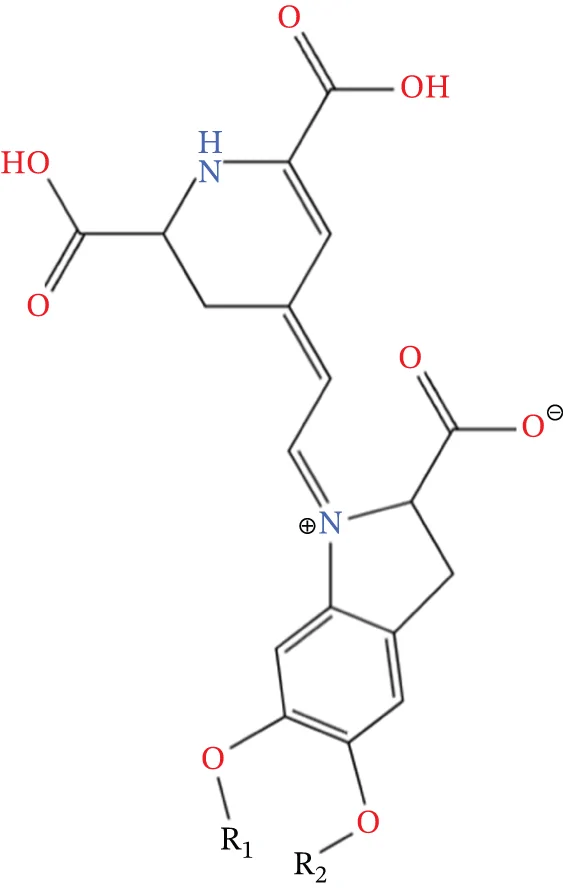
Basic structure of betacyanins. The R groups in their respective structures give rise to different types of betacyanin.

**Figure 8:**
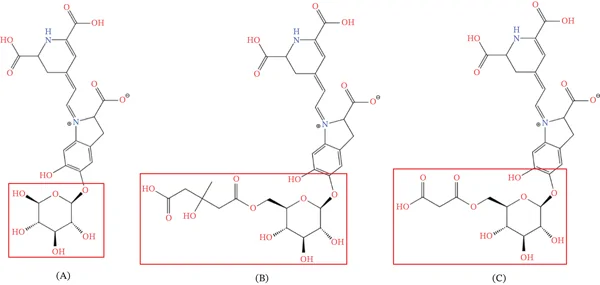
Structures of (A) betanin, (B) hylocerenin, and (C) phyllocactin. The red boxes highlight their different R groups.

When IL‐1*β* is cleaved from its precursor form, the C‐terminal fragment (amino acid residues 113–271) is released to become the active mature form [53]. Of these, residues 119–265 represent the IL‐1 domain (SMART, UniProt ID: P01584). The literature has also identified amino acid residues 117–267 of IL‐1*β* (after accounting for the cleavage of the N‐terminal region to release the mature form) as crucial for biological activity [54–57]. When complexed with its receptor, IL‐1R1, residues 13–17, 110–115, 120–125, 239–245, 256, 257, 293–299, and 302–311 of IL‐1*β* are binding elements that interact with all three immunoglobulin‐like domains of IL‐1R1 [58]. All four betacyanins formed hydrogen bond interactions in this crucial region of IL‐1*β* (PDB Code: 9ILB), which may have affected the binding activity of IL‐1*β* to its receptor, leading to potential changes in the downstream signaling activity in which IL‐1*β* is involved. For IL‐1R1 (PDB Code: 1IRA), according to SMART (UniProt ID: P14778), the three immunoglobulin‐like domains span amino acid residues 28–108, 129–215, and 233–330. Based on Table 4, the four betacyanins targeted primarily the region between residues 129 and 215, which appears to be particularly crucial, as amino acid loops 164–173 and 202–214 have been identified as crucial for agonistic binding between IL‐1*β* and IL‐1R1 [57]. Specifically, there were two hydrogen bond formations between phyllocactin and the amino acid loop 164–173, specifically LEU167 and LEU169. As mentioned previously, since the three immunoglobulin‐like domains of IL‐1R1 bind IL‐1*β* during binding, interactions in this region formed by the four betacyanins are speculated to affect IL‐1*β* activity.

IL‐6 has three binding sites formed by four helices, namely, Helices A, B, C, and D. Site 2 binds with the D2 and D3 domains of gp130, and Site 3 binds to the D1 domain of gp130. Together with the binding of Site 1 to IL‐6R, it forms a functional hexameric complex [59]. The IL‐6 domain was identified as residues 57–210 (SMART, UniProt ID: P05231). The C‐terminal region found in Helix D (residues 166–183) appears to be of interest for site mutagenesis of amino acids in this region, as it either exerted changes in binding affinity to IL‐6R, changes in biological activity in vitro, or both [60]. Of these, crucial amino acid residues were identified to be Q174, R178, and R181, where site mutagenesis analysis of IL‐6 has shown that mutants Q174A, R178A, and R181A/V exhibited altered binding energy compared with wild‐type IL‐6 [60]. Additionally, conserved residues F173, R179, and R182 are part of the active site architecture, and mutations at these sites cause IL‐6 to lose significant biological activity [61]. Its receptor, IL‐6R, has two domains: an immunoglobulin C2 type domain (residues 38–103) and a fibronectin Type 3 domain (residues 216–301), which were identified through SMART (UniProt ID: P08887). While the fibronectin Type 3 domain is usually associated with cellular proliferation, adhesion, and migration [62], it is found that the fibronectin Type 3 domain in gp130, in the context of IL‐6, does not play a role in recognition, but is instead crucial in receptor activation, as this domain raises the ligand‐binding section of the receptor higher off the cell surface [63]. With respect to the C‐terminal region (residues 166–183) of IL‐6 (PDB Code: 1ALU), whereas none of the betacyanins formed hydrogen bonds with certain critical residues such as Q174, R178, and R181, several hydrogen bonds were formed in the crucial region between residues 166 and 183 (Table 3), which is speculated to have altered IL‐6 biological activities. On the other hand, Table 4 shows that the four betacyanins appear to target the fibronectin Type 3 domain of IL‐6R (PDB Code: 8QY5), which may indirectly disrupt or promote IL‐6 recognition by altering the receptor′s elevation above the cell surface.

In TNF‐*α*, residues 88–233 constitute the TNF domain (SMART, UniProt ID: P01375). After trimerization, TNF‐*α* binds to its receptor, TNFR1, leading to downstream activation of signaling pathways [64]. In the lowest binding energy model shown in Table 3, all four betacyanins formed multiple hydrogen bonds in the TNF domain upon docking to TNF‐*α* (PDB Code: 6OP0). Four TNFR domains (residues 44–81, 84–125, 127–166, and 168–195), as well as the DEATH domain (345–441), were identified in the TNFR1 receptor from SMART (UniProt ID: P19438). The four TNFR domains, also known as cysteine‐rich domains, appear to be cysteine‐rich repeats responsible for ligand binding [65]. Apart from hylocerenin, the other betacyanins docked against TNFR1 (PDB Code: 7KPB) targeted the two TNFR domains between residues 84 and 81 and between 127 and 166 (Table 4). Hylocerenin appeared to have formed four hydrogen bonds in the region between 44 and 81 (Table 4). Based on observations of both TNF‐*α* and TNFR1, betacyanins appeared to target crucial binding domains in both the protein and the receptor, potentially affecting TNF‐*α* trimerization or its binding to its receptor.

The SH2 domain of SOCS3 encompasses the region between amino acid residues 44 and 133 (SMART, UniProt ID: O14543) which is crucial for regulating cytokine signal transduction, with gp130 as its main receptor, leading to downstream cytokine regulation [66, 67]. Table 3 shows that all four betacyanins formed hydrogen bonds in the SH2 domain of SOCS3 (PDB Code: 2HMH), which is suspected to have affected the binding of SOCS3 to gp130. Regarding gp130, it is established that two tyrosine residues, TYR757 and TYR759, are critical for SOCS3 to bind to and exert its inhibitory effects [68, 69]. However, there are currently no experimental structures from SWISS‐MODEL that cover TYR757 and TYR759 (UniProt ID: P40189). Therefore, no docking was performed between the four betacyanins and gp130.

## 4. Conclusion

This study demonstrated that 50 *μ*g/mL of betacyanin fraction was able to modulate key proinflammatory cytokines such as IL‐1*β*, IL‐6, and TNF‐*α* in an unexpected and unreported manner that might contribute to the host cellular defense against IAV after up to 72 h, resulting in the cell′s survival against the virus. Based on current betacyanin literature, betacyanin treatment was expected to downregulate cytokine levels. However, the present results suggest that betacyanin upregulated these cytokine levels during IAV infection instead, potentially contributing to viral clearance. While current literature also indicates that overexpression of cytokines during IAV infection causes significant damage to the host cell, these cytokines are also paramount in mounting a significant defense against the virus as part of the innate immunity. To the authors′ best knowledge, this is the first time betacyanins have demonstrated the ability to upregulate proinflammatory cytokines. Additionally, the mRNA expression levels of a key host factor that the virus hijacks, SOCS3, were downregulated by betacyanins after up to 72 h, further contributing to the cell′s survival. Molecular docking was also performed on selected host factors, and it was determined that all four betacyanins were able to bind and interact with IL‐1*β*, IL‐6, TNF‐*α*, and SOCS3, as well as their respective receptors. Although it is postulated that the upregulation of IL‐1*β*, IL‐6, and TNF‐*α* contributed to the survival of the cell, it would be beneficial to investigate the immunological aspect of the cell, such as the secretion of Type 1 and 3 IFNs and chemokines, as it is speculated that the upregulation of cytokines contributed to the antiviral defense mechanism. It would also be beneficial to complement the observed changes in mRNA expression levels with an analysis of protein expression levels to validate that the modulated mRNA expression levels translate into protein activity. The use of a primary cell line, such as bronchial/tracheal epithelial cells, would provide a more accurate physiological representation of the cytokine expression as compared with the immortalized A549 cell line. Furthermore, adopting a clinically relevant strain, such as an A(H1N1)pdm09 strain, would better reflect cytokine expression observed in clinical settings.

## Author Contributions

Chie Min Lim: data curation, formal analysis, investigation, methodology, and writing—original draft; Nurulfiza Mat Isa: resources and writing—review and editing; Abdul Rahman Omar: writing—review and editing; Wee Sim Choo: conceptualization, funding acquisition, project administration, resources, supervision, and writing—review and editing.

## Funding

This work was supported by the Ministry of Higher Education, Malaysia (10.13039/501100003093, FRGS/1/2020/SKK0/MUSM/02/1). Open access publishing facilitated by Monash University, as part of the Wiley ‐ Monash University agreement via the Council of Australasian University Librarians.

## Conflicts of Interest

The authors declare no conflicts of interest.

## Supporting information


**Supporting Information** Additional supporting information can be found online in the Supporting Information section. Table S1: Quantitative PCR (qPCR) primer sequences. Figure S1: Representative melting curve of the housekeeping gene, *β*‐actin, as well as all four other host factors, namely, IL‐1*β*, IL‐6, TNF‐*α*, and SOCS3.

## Data Availability

The data that support the findings of this study are available from the corresponding author upon reasonable request.
