# Supplementary material for: Red Pitahaya Betacyanins Selectively Upregulate Proinflammatory Cytokines In Vitro During Influenza A Virus Infection
Source: Int J Microbiol. 2026 Oct 22;2026:4807321. doi: 10.1155/ijm/4807321 (PMC13689544; doi:10.1155/ijm/4807321)
Supplement: Supplementary file 1 [file IJM-2026-4807321-s001.docx]

**Table S1.** Quantitative PCR (qPCR) primer sequences

| **Target** | **Forward** | **Reverse** |
| --- | --- | --- |
| Human IL-1β mRNA | 5′ – TCT GGG ATT CTC TTC AGC CA – 3′ | 5′ – TCT GTT TAG GGC CAT CAG CTT – 3′ |
| Human IL-6 mRNA | 5′ – GAC AGC CAC TCA CCT CTT CA – 3′ | 5′ – GCC TCT TTG CTG CTT TCA CA – 3′ |
| Human TNF-α mRNA | 5′ – GCA ACA AGA CCA CCA CTT CG – 3′ | 5′ – GAT CAA AGC TGT AGG CCC CA – 3′ |
| Human SOCS3 mRNA [1] | 5′ – GGA GTT CCT GGA CCA GTA CG – 3′ | 5′ – TTC TTG TGC TTG TGC CAT GT – 3′ |
| Human β-actin | 5’ – CTT CGC GGG CGA CGA T – 3’ | 5’ – CCA CAT AGG AAT CCT TCT GAC C – 3’ |

**References**

1. Ehlting C, Lai WS, Schaper F, BrenndöRfer ED, Matthes R-J, Heinrich PC, Ludwig S, Blackshear PJ, Gaestel M, HäUssinger D & Bode JG. Regulation of suppressor of cytokine signaling 3 (SOCS3) mRNA stability by TNF-α involves activation of the MKK6/p38MAPK/MK2 cascade1. J immunol 2007;178:2813-2826.

**Figure S1.** Representative melting curve of the housekeeping gene, β-actin, as well as all four other host factors, namely IL-1β, IL-6, TNF-α, and SOCS3.
